# A Randomized Clinical Trial Evaluating the Effect of an Oral Calcium Bolus Supplementation Strategy in Postpartum Jersey Cows on Mastitis, Culling, Milk Production, and Reproductive Performance

**DOI:** 10.3390/ani11123361

**Published:** 2021-11-24

**Authors:** Paulo R. Menta, Leticia Fernandes, Diego Poit, Maria Luiza Celestino, Vinicius S. Machado, Rafael C. Neves

**Affiliations:** 1 Department of Veterinary Sciences, College of Agricultural Sciences and Natural Resources, Texas Tech University, Lubbock, TX 79409, USA; paulo.menta@ttu.edu (P.R.M.); lfernandes@lelly.com (L.F.); diego.poit@usp.br (D.P.); mkcelestino@gmail.com (M.L.C.); Vinicius.Machado@ttu.edu (V.S.M.); 2 Department of Veterinary Clinical Sciences, College of Veterinary Medicine, Purdue University, West Lafayette, IN 47907, USA

**Keywords:** dairy cow, Jersey, oral calcium bolus, calcium, hypocalcemia, mastitis, culling, milk production, reproduction

## Abstract

**Simple Summary:**

The time around parturition is a challenging period in the lactation cycle of high-yielding dairy cows as it is characterized by several endocrine, metabolic, and physiological changes. Among those challenges, calcium demands are rapidly increased to support colostrogenesis and lactogenesis during a time of reduced dry matter intake; invariably, some cows may suffer from clinical and subclinical hypocalcemia. Oral calcium supplementation is a common preventative strategy adopted in the postpartum of high-yielding dairy cows to minimize the negative impact of suboptimal blood calcium concentration during this period. Despite a great number of studies evaluating the effects of oral calcium supplementation in postpartum Holstein cows, very limited information is available for the Jersey breed. This study aimed to evaluate the effect of an oral Ca supplementation strategy in the first 24 h after parturition on health and production outcomes in multiparous Jersey cows. Overall, treatment did not improve milk production or reproductive performance compared to control cows. Additionally, treatment had no effect on early lactation culling. A tendency for a minor impact on the odds of mastitis was evident though it depended on the parity of the cows.

**Abstract:**

The objectives of this study were to evaluate the effects of a postpartum oral calcium supplementation strategy in multiparous Jersey cows on (1) the odds of clinical mastitis in the first 60 days in milk (DIM); (2) the odds of culling up to 60 DIM; (3) the risk of pregnancy in the first 150 DIM; (4) milk production in the first 15 weeks of lactation. A randomized clinical trial was performed in a dairy herd located in west Texas, United States. A total of 809 cows were used in the final analyses. Overall, postpartum oral calcium supplementation did not influence milk production, reproductive performance, or culling. Among second parity cows, oral calcium supplementation tended to decrease the odds of clinical mastitis in the first 60 DIM compared to controls; however, no differences were observed for cows in parities three and greater. To date, data evaluating the effect of postpartum oral calcium supplementation in multiparous Jersey cows are limited. In our study, oral calcium supplementation tended to reduce clinical mastitis in second parity cows. No positive benefits based on the reduction of culling, and improvement of milk production and reproductive performance were evident for the herd included in this study.

## 1. Introduction

The time around parturition is a challenging period in the lactation cycle of high-yielding dairy cows. Maladaptive responses to the increased nutrient requirements and stressors in the transition to lactation can impact lactation performance. Daily calcium (Ca) requirements increase approximately 1.6 times from late prepartum to early postpartum [1,2], highlighting the importance of Ca in the transition of the non-lactating to lactating state; those changes are due to the onset of colostrogenesis and lactogenesis. Invariably, suboptimum Ca concentration is common within 12 to 24 h after calving and can extend to a few days in lactation [3,4,5]. In order to meet the increased Ca requirements, the activation of homeostatic and homeorhetic mechanisms such as renal reabsorption, bone resorption, and intestinal absorption are essential [1]. Despite that, some cows fail in this process and may experience clinical or subclinical hypocalcemia in the postpartum period.

Clinical hypocalcemia (CH) is characterized by an acute reduction in blood Ca concentration below 1.4 mmol/L, and it is usually detected within 72 h after parturition [6]. Improvement of nutritional strategies applied in the prepartum period has helped to reduce CH over the years [7,8]. As an example, an acidogenic diet applied in the prepartum as a preventative dietary strategy against CH became popular and is proven to be an effective method [9,10,11].

Subclinical hypocalcemia (SCH) has caught more attention over the last decade because of its difficulty to diagnose and high prevalence among U.S. and European herds [7,12,13]. Even though it does not present evident signs, it has an important economic impact on dairy farms due to negative effects on dry matter intake at the beginning of lactation [14]. In addition, SCH has been associated with negative health outcomes such as retained placenta, metritis, impaired reproductive performance [15], displaced abomasum [16], increased culling rate [17], and impaired immune function [18]. Unfortunately, acidogenic diets have not been demonstrated to be as effective for SCH prevention as for CH [7]. Therefore, strategies to mitigate the potential effects of SCH via postpartum oral Ca supplementation are still widely adopted. In the U.S. for instance, 80% of the large farms used some combination of injectable, drench, or oral Ca as a preventative strategy to postpartum diseases [19]. The use of oral Ca supplementation increases blood Ca concentration [20], though the data evaluating the benefits of oral Ca are inconsistent across cows; some groups can benefit from it (e.g., high milk producers, lame cows) while others do not (e.g., first parity cows) [8,21]. Data evaluating the effect of postpartum oral Ca supplementation in health and production outcomes are limited for the Jersey breed.

The current literature is limited when evaluating the effect of postpartum oral Ca supplementation in Jersey cows, and results obtained in studies performed in Holstein cows may not directly apply to the Jersey breed. Jersey cows are well known for having an increased susceptibility to CH [6,22]; they mobilize more Ca into milk [23] and have a lower abundance of 1,25-dihidroxyvitamin D3 receptors in their intestine [22] which can contribute to a reduced dietary intestinal Ca absorption capacity. A study evaluating the effect of postpartum oral Ca supplementation for Jersey and Jersey–Holstein crossbreeds demonstrated an increase in blood Ca levels and a lower prevalence of SCH in treated cows [24]. The effect of postpartum oral Ca supplementation on milk yield was conditional to cow-level factors such as previous lactation length and calving locomotion score [25]. More studies evaluating the benefits of postpartum oral Ca supplementation in health, production, and reproductive outcomes for purebred Jersey cows are warranted.

The objective of our study was to determine the effect of an oral Ca supplementation strategy applied to multiparous Jersey cows (one bolus given soon after parturition followed by a second bolus 12–24 h after) on health outcomes, reproductive performance, and milk production. We hypothesized that postpartum oral Ca supplementation would decrease the odds of clinical diseases and improve milk production and reproductive performance. Our study was designed to mimic how postpartum Ca supplementation is commonly performed in U.S. dairy farms (blanket treatment) to better inform veterinarians and producers on the potential benefits of this strategy for the Jersey breed.

## 2. Materials and Methods

### 2.1. Study Design and Data Collection

A randomized clinical trial was conducted from July 2018 to April 2019 at a commercial dairy farm in west Texas milking 3800 Jersey and Jersey–Holstein crossbred cows. A sample size calculation determined that at least 788 cows were necessary for a study with 80% power and significant differences declared at α = 0.05 assuming an expected difference of at least 1.0 kg of milk per day between treated and control cows, with a daily milk production SD of each group being of 5.0 kg, and an equal number of cows among groups (k = 1).

A more detailed description of herd demographics, average milk production, reproductive program used, and total mixed ration offered to the close-up and fresh cows can be found in Menta et al. (2021) [5]. Of note, prepartum cows were fed a negative dietary cation-anion diet. Cow enrollment occurred from July to December 2018; for this study, only purebred multiparous Jersey cows entering their 2nd or greater lactation were randomly assigned to the treatment or control group. The farm was provided with randomized enrollment sheets blocked by parity groups (2nd vs. 3rd and greater) prepared by the research group beforehand, and cows were sequentially enrolled by calving date and time by farm personnel. The treated cows received two doses of a commercial oral Ca bolus (Bovikalc^®^, Boehringer Ingelheim Vetmedica, Inc., St. Joseph, MO, USA) containing calcium chloride and calcium sulfate (43 g of Ca per bolus); the first dose was administered by farm personnel within 1 h after calving in a chute at the maternity area. The 2nd dose of the Ca bolus was administered by the research personnel the day after parturition, which occurred approximately 21 h and 20 min after calving (SD = 6 h) while cows were restrained in headlocks after the morning milking. In total, 852 multiparous cows were enrolled. Cows that had twins or that aborted (gestation length < 260 days) were not included in the study.

Milk yield was recorded by the research group weekly. Milk records were logged using a manually portable device (Pocket CowCard, Valley Agricultural Software, Tulare, CA, USA) which was automatically downloaded into the herd management software (DairyComp 305, Valley Agricultural Software, Tulare, CA, USA). Disease event data were recorded by farm personnel in DairyComp 305 using disease definition protocols accorded before the start of the study. Retained placenta (RP) was defined as the failure to expel fetal membranes within 12 h of parturition [26]. Clinical hypocalcemia (CH) was defined as a cow that was recumbent within 72 h of parturition accompanied by cool extremities and reduced ruminal contractions [27]. Displaced abomasum (DA) was defined as a classical resonant sound (indicating the presence of gas) during concurrent auscultation and percussion in an imaginary line from the coxae to the olecranon of the left/right flank region [27]. Mastitis was defined as any abnormalities during milk secretion, such as in color and clots, with or without local visible signs of inflammation in the udder [27]. Dystocia was defined as any human intervention during parturition. Body condition score (BCS) was evaluated at 4 DIM by the first author, who was blinded to treatment groups, using a 5-point scale [28].

### 2.2. Statistical Analyses

Cow-level data including health events, reproduction, and milk production were extracted from DairyComp 305 into Microsoft Excel (Microsoft Corp., Redmond, WA, USA) before statistical analyses in SAS v9.4 (SAS Institute Inc., Cary, NC, USA). Descriptive statistics were performed using the UNIVARIATE, FREQ, and MEANS procedures. For the binary and continuous outcomes, the GLIMMIX and MIXED procedures were used, respectively. Continuous data were evaluated for distribution of the residuals and homogeneity of variance after model fitting.

A binary variable representing calving-related problem(s) was created to characterize cows that suffered from dystocia (i.e., any cow requiring human intervention for calf extraction) and(or) had a stillbirth. Body condition score was categorized to represent thin (BCS ≤ 2.75), normal (BCS between 3.0 and up to 3.5), and over-conditioned cows (BCS ≥ 3.75). Parity was dichotomized to represent 2nd versus ≥3rd parities. Calving season was considered as a dichotomous variable (warmer months: cows calving from July to September 22, 2018; cooler months: calvings occurring from September 23 to December 2018). Average temperature for the warmer and cooler months were 26.4 °C (SD: 3.4 °C) and 11.7 °C (SD: 6.8 °C), respectively, according to temperature records retrieved from the National Weather Service database (National Oceanic and Administrative Association, United States Department of Commerce, Silver Spring, MD, USA) and based on the herd’s ZIP code.

Univariable analyses were performed to screen variables associated with the outcomes at *p* ≤ 0.20 before inclusion to the multivariable models. A manual backward stepwise selection procedure was used, and variables at *p* ≤ 0.05 were retained as main effects. If a variable caused more than 20% change in one or more estimates, it was maintained as a confounder. The effect of treatment was forced in all models as it was the main predictor of interest. Parity was considered a confounder a priori and, therefore, included in all models regardless of the significance value. Potential 2-way interactions were tested in the final models between significant variables and the predictor of interest. Potential predictors considered in the models were calving-related problems, calving season, BCS score (mastitis, culling, pregnancy to 150 DIM, and milk yield models), mastitis up to 60 DIM (culling model), weekly milk test number, and previous gestation length (milk production model). As the studied herd did not participate in a Dairy Herd Improvement program, previous lactation 305 mature equivalent data were not available to be used as a covariate in the milk production model.

For health parameters, the odds of mastitis in the first 60 DIM was the sole outcome evaluated. Clinical hypocalcemia, RP, and DA had very low incidences during the study. Metritis diagnosis was not consistently performed across all cows in the herd and was, therefore, not evaluated. To evaluate the effect of treatment on the odds of mastitis and culling, a multivariable logistic regression model was fitted to the data using the GLIMMIX procedure. The LSMEANS option was used to calculate the least square means and standard error of the mean to report proportions. Cox’s proportional hazard model was fitted using the PHREG procedure for pregnancy up to 150 DIM. Cows were left-censored if not diagnosed as being pregnant before being culled or if they died within the survival time. A generalized linear mixed model was fitted for the milk production data using the MIXED procedure while accounting for the repeated measurements within cow. To appropriately account for within-cow correlation, the error term was modeled by imposing a Toeplitz heterogenous covariance structure as it yielded the smallest Akaike’s information criterion from the ones tested. For all models described above, statistical significance was declared if *p* ≤ 0.05, and a tendency was considered if 0.05 < *p* < 0.1.

## 3. Results

A total of 852 cows (treatment: 418 and control: 434) were initially enrolled in the study. Of the total number of enrolled cows, 27 and 16 cows in the treatment and control groups, respectively, were excluded. Exclusion of cows in the treatment group were for the following reasons: 3 cows were first parity, 9 were Jersey–Holstein crosses, 3 cows were diagnosed with mastitis at 0 DIM, and 12 did not receive their second bolus. Exclusion of cows in the control group were for the following reasons: two cows were first parity, four were Jersey–Holstein crosses, 3 cows were diagnosed with mastitis at 0 DIM, one cow had uterine prolapse, and six were treated with a Ca bolus by mistake. Thus, 809 cows (treatment: 391 and control: 418) remained in the experiment for final analyses; 310 (38%) were of second parity and 499 (62%) of third and greater parities. Descriptive statistics data depicting incidences of early lactation disorders for the cows included in the final analyses can be found in Table 1.

### 3.1. Mastitis

A parity by treatment group interaction was found in the final model evaluating the odds of clinical mastitis in the first 60 DIM (*p* = 0.02). Among second parity cows, treatment tended to reduce the odds of clinical mastitis (OR = 0.49; *p* = 0.07) compared to controls. The same was not true among third and greater parity cows; there was no difference in the odds of clinical mastitis when treated cows were compared to controls (OR = 1.45; *p* = 0.49). The other variable retained in the final model was the effect of a calving-related problem (*p* = 0.49). Table 2 presents the odds ratio for the parity by treatment group interaction for the mastitis model.

### 3.2. Culling

Treatment was not associated with the odds of culling in the first 60 DIM (*p* = 0.72). Variables remaining in the final model were parity (*p* = 0.001), and calving-related problems (*p* = 0.01). Culling incidence in the first 60 DIM for the treated and controls cows were 8.7 and 9.3%, respectively. Table 3 presents the final logistic regression model evaluating the effect of postpartum oral Ca supplementation on the odds of culling in the first 60 DIM.

### 3.3. Reproductive Performance

The Cox proportional hazards model revealed no effect of treatment on time to pregnancy (*p* = 0.67). Other variables retained in the final model were the effect of parity (*p* = 0.91) and calving-related problems (*p* = 0.16). The effect of calving season violated the assumption of proportional hazards over time and was included in the STRATA statement. Table 4 presents the final Cox proportional hazards model.

### 3.4. Milk Production

Treatment had no effect on milk production for the first 15 weeks of lactation (*p* = 0.73). On average, treated and control cows produced 33.5 kg/d (SEM ± 0.59) and 33.3 kg/d (SEM ± 0.36), respectively. Other variables included in the model were the effect of parity (*p* = 0.22), BCS score (*p* = 0.25), calving season (*p* = 0.02), gestation length (*p* = 0.0001), and week of milk measurement (*p* < 0.0001). The final milk model is presented in Table 5.

## 4. Discussion

Currently, studies evaluating the effect of postpartum oral Ca supplementation focusing on health, production, and reproduction in purebred Jersey cows are lacking. The objectives of this study were to evaluate the effect of an oral postpartum Ca supplementation strategy comprised of 43 g of calcium salts administered within 1 h after parturition, followed by a second dose the day after parturition, on the odds of clinical mastitis and culling within 60 DIM, as well as on milk production and reproductive efficiency in multiparous Jersey cows. Subclinical hypocalcemia is a risk factor for early lactation diseases, impaired reproduction, and culling in dairy herds [21,29,30]. It is a common belief among dairy farmers that oral Ca supplementation postpartum decreases the incidence of some postpartum diseases, and can potentially reduce culling and improve milk production and reproductive efficiency. Our results showed no effects of the oral Ca supplementation strategy in multiparous postpartum Jersey cows on early-lactation culling, milk production, or reproductive efficiency. There was a tendency for oral Ca supplementation in second parity cows to have reduced odds of mastitis in the first 60 DIM; the same was not true for third and greater parity cows.

The use of postpartum oral Ca supplementation in Holstein cows has been associated with improved cow performance. A study done comparing the effect of two different doses (43 and 86 g) of oral Ca supplementation showed that both treatments were able to increase the concentration of ionized Ca in the blood [31]. Additionally, the same study showed that oral Ca supplementation improved reproductive performance in multiparous cows only, while having a detrimental effect in primiparous cows. When oral Ca supplementation is administrated to specific groups of cows, it seems to have a more relevant impact on health [8,32]. Leno et al. (2018) demonstrated in Holstein cows that a single dose of Ca oral supplementation within 24 h postpartum improved health status for cows of greater parity and BCS, and lame cows [33]. Additionally, a recent study done in Jersey and Jersey–Holstein crosses showed that the effects of the treatment were dependent on cow-level factors and had minimal impacts on group-level assessments [25]. A limitation of our study is the absence of lameness information (data not collected by our group) and previous lactation milk yield (herd not participating in Dairy Herd Improvement program) which limited our ability to explore some cow-level dependent effects.

We evaluated the effect of oral Ca supplementation on the odds of clinical mastitis within 60 days after parturition and found that the results depended on parity; among second parity cows, treatment tended to decrease the odds of mastitis. Suboptimum Ca concentration in the blood is associated with a reduced concentration of Ca in the cytosol of leukocytes and a reduced immune response [34]. A study done on Jersey–Holstein crosses demonstrated improved neutrophil phagocytosis and oxidative burst when cows were supplemented with two oral Ca (50g) boluses in the first 24 h after parturition [35]. Domino et al. (2017) found that the postpartum administration of Ca boluses decreased the risk of mastitis in multiparous Holstein cows within the group of cows with a high relative herd rank only (a metric that was based on previous lactation milk production) [36]. However, other studies evaluating the effect of oral Ca boluses on health outcomes reported no effect of treatment on the risk of mastitis [8,31].

Treatment had no impact on the odds of culling in the first 60 DIM. Milk production and reproduction are two main factors affecting culling in dairy herds [37,38]. No treatment effect was found for milk production in the first 15 weeks of lactation or reproductive efficiency up to 150 DIM; therefore, it is not surprising that oral Ca supplementation also did not have any impact on the odds of culling. We are unaware of any studies reporting an association of postpartum oral Ca supplementation and decreased culling.

No effect of treatment on time to pregnancy was found. The current literature does not report any beneficial effect of postpartum prophylactic Ca administration on reproductive performance in Holstein and Jersey cows as a blanket support therapy [25,39,40]; Martinez et al. (2016) administered Ca boluses to Holsteins cows considered to be of high and low risk to develop metritis in a randomized block design and found a benefic impact on reproduction outcomes for cows in the high-risk metritis block [31]. No effect of oral Ca treatment was found in pregnancy at first service in a previous Jersey study [25]. The literature is inconsistent when evaluating low concentrations of Ca in the blood as a risk factor for impaired reproduction [5,30,37,41]. While we did not measure serum Ca dynamics in early postpartum, Valldecabres et al. (2021) demonstrated that Jersey cows with Ca ≤ 1.94 mmol/L within the first hours postpartum had decreased risk of pregnancy [41]. On the other hand, Menta et al. (2021) demonstrated no association of blood Ca levels in the first 3 d postpartum and pregnancy to first service [5]. Although we did not find positive effects of oral Ca boluses in Jersey cows with most outcomes evaluated, we cannot rule out that some cow subpopulations could benefit from this strategy. One potential limitation of our study is that we did not measure blood Ca concentrations following oral Ca supplementation to confirm the rise in systemic Ca levels post-treatment. However, we used a commercial product that has been used in previous studies and proven to increase blood Ca concentrations [31,36].

No effect of postpartum oral Ca was found on milk yield in the first 15 weeks after lactation. This result agrees with previous research conducted in Holstein cows [33,42,43]. Oetzel and Miller (2012) found a positive effect of Ca boluses for lame and high-producing cows only [8]. Our results are consistent with those of Valldecabres and Silva-del-Rio (2021), which was conducted in a population of Jersey and Jersey–Holstein crossbred cows [25]. A recent study done by our research group evaluated the association of Ca concentration in the first 3 DIM with milk production and found that Jersey cows with reduced Ca concentrations at 1 (≤1.84 mmol/L) and 2 DIM (≤2.04 mmol/L) had increased milk yield in the first 9 weeks after calving [5]. Therefore, further research evaluating how different oral Ca supplementation protocols that vary the timing of bolus administration relative to calving are warranted.

## 5. Conclusions

In summary, our randomized clinical trial demonstrated that prophylactic postpartum Ca supplementation to multiparous Jersey cows had no effects on culling, milk yield, and reproduction. Second parity cows that were supplemented with oral Ca boluses tended to have reduced odds of mastitis compared to non-supplemented cows; however, no treatment effect was evident for third and greater parity cows. This is one of the few large randomized clinical trials evaluating the effects of postpartum Ca supplementation in Jersey cows. Our data do not support blanket oral Ca supplementation in Jersey cows as the effects were minimal to none; however, targeted oral Ca supplementation for subpopulations of cows and at different times relative to parturition remain to be investigated.

## Figures and Tables

**Table 1:** Early lactation disorders in a randomized clinical trial evaluating the effects of an oral postpartum calcium supplementation strategy in multiparous Jersey cows (*n* = 809) in a dairy in west TX.

Item	Control (*n* = 418)		Treatment (*n* = 391)	
	Parity 2	Parity ≥ 3	Parity2	Parity ≥ 3
Dystocia	4	8	6	15
Stillbirth	4	10	4	5
Clinical hypocalcemia ^1^	1	4	0	8
Retained placenta	0	3	0	1
Left displaced abomasum	1	1	0	1

^1^ Clinical hypocalcemia diagnosis occurred within 24 h after calving.

**Table 2:** Odds of clinical mastitis in the first 60 DIM by parity in a randomized clinical trial evaluating the effect of an oral postpartum calcium supplementation strategy in multiparous Jersey cows (*n* = 809) in a dairy in west TX.

Comparison	Estimate	Odds Ratio	95% CI ^1^	*p*-Value
Parity 2: treatment versus control	−0.71	0.49	0.18–1.33	0.07
Parity ≥ 3: treatment versus control	0.37	1.45	0.74–2.83	0.49

^1^ Confidence interval.

**Table 3:** Final logistic regression model evaluating the effect of an oral postpartum calcium supplementation strategy with culling in the first 60 DIM in a randomized clinical trial in multiparous Jersey cows (*n* = 809) in a dairy in west TX.

Variable	Estimate	SE ^1^	*p*-Value
Intercept	−3.07	0.30	<0.001
Postpartum Ca supplementation			
Control	Ref ^2^	–	–
Treatment	−0.09	0.25	0.72
Parity			
2	Ref ^2^	–	–
≥3	1.01	0.31	0.001
Calving problem ^3^			
No	Ref ^1^	–	–
Yes	0.94	0.38	0.01

^1^ Standard error. ^2^ Reference category (i.e., the value to which the variable level is being compared to while controlling for the effect of the other predictors in the model). ^3^ Calving problem: variable representing cows that suffered from dystocia and(or) that had a stillbirth.

**Table 4:** Cox proportional hazards model evaluating the effect of an oral postpartum calcium supplementation strategy with reproductive efficiency in a randomized clinical trial in multiparous Jersey cows (*n* = 809) in a dairy in west TX.

Variable	Estimate	SE ^1^	*p*-Value	Hazard Ratio	Hazard Ratio CI
Postpartum Ca supplementation					
Control	Ref ^2^	–	–	–	–
Treatment	0.04	0.10	0.67	1.04	0.86–1.27
Parity					
2	Ref ^2^	–	–	–	–
≥3	−0.01	0.10	0.91	0.99	0.8–1.20
Calving problem ^3^					
No	Ref ^2^	–	–	–	–
Yes	−0.37	0.26	0.16	0.69	0.41–1

^1^ Standard error. ^2^ Reference category (i.e., the value to which the variable level is being compared to while controlling for the effect of the other predictors in the model). ^3^ Calving problem: variable representing cows that suffered from dystocia and(or) that had a stillbirth.

**Table 5:** Final linear mixed model evaluating the effect of an oral postpartum calcium supplementation strategy with milk production within 15 weeks of lactation in a randomized clinical trial in multiparous Jersey cows (*n* = 809) in a dairy in west TX.

Variable	Estimate	SE ^1^	*p*-Value
Intercept	−9.62	10.83	0.35
Postpartum Ca supplementation			
Control	Ref ^2^	–	–
Treatment	0.24	0.69	0.73
Parity			
2	Ref ^2^	–	–
≥3	0.50	0.41	0.22
Test number	–	–	<0.001
Calving season ^3^			
Warm	Ref ^2^	–	–
Cool	−0.97	0.40	0.02
Gestation length (days)	0.15	0.04	<0.001
Body condition score			
Thin	Ref ^2^	–	–
Normal	0.76	0.72	0.29
Over-conditioned	1.64	0.99	0.10

^1^ Standard error. ^2^ Reference category (i.e., the value to which the variable level is being compared to while controlling for the effect of the other predictors in the model). ^3^ Calving season (warm: cows calving from 19 July to 22 September 2018; cool: cows calving from 23 September to 9 December 2018).

## Data Availability

Data not available due to participating farm restrictions.
